# The Yield of Active Tuberculosis Disease and Latent Tuberculosis Infection in Tuberculosis Household Contacts Investigated Using Chest X-ray in Yogyakarta Province, Indonesia

**DOI:** 10.3390/tropicalmed9020034

**Published:** 2024-01-31

**Authors:** Betty Nababan, Rina Triasih, Geoffrey Chan, Bintari Dwihardiani, Arif Hidayat, Setyogati C. Dewi, Lana Unwanah, Arif Mustofa, Philipp du Cros

**Affiliations:** 1 Centre for Tropical Medicine, Faculty of Medicine, Public Health and Nursing, Gadjah Mada University, Sleman, Yogyakarta 55281, Indonesia; 2 Department of Pediatric, Faculty of Medicine, Public Health and Nursing, Gadjah Mada University, Dr. Sardjito Hospital, Sleman, Yogyakarta 55281, Indonesia; 3 TB Elimination and Implementation Science Working Group, Burnet Institute, Melbourne, VIC 3004, Australia; 4 Yogyakarta City Health Office, Yogyakarta, Yogyakarta 55165, Indonesia; 5 Kulon Progo District Health Office, Yogyakarta, Yogyakarta 55165, Indonesia

**Keywords:** household contact, contact investigation, active TB, latent TB infection, factors

## Abstract

In Indonesia, the implementation of tuberculosis (TB) contact investigation is limited, with low detection rates. We report the yield of and risk factors for TB disease and infection for household contacts (HHCs) investigated using chest X-ray (CXR) screening. We identified HHCs aged five years and above of bacteriologically confirmed index cases from 2018 to 2022 in Yogyakarta City and Kulon Progo. All HHCs were offered screening for TB symptoms; TB infection testing with either tuberculin skin testing or interferon gamma release assay; and referral for CXR. Sputum from those with symptoms or CXR suggestive of TB was tested with Xpert MTB/RIF. Risk factors for active TB disease and latent TB infection (LTBI) were identified by logistic regression models. We screened 2857 HHCs for TB between June 2020 and December 2022, with 68 (2.4%) diagnosed with active TB. Of 2621 HHCs eligible for LTBI investigation, 1083 (45.7%) were diagnosed with LTBI. The factors associated with active TB were age, being underweight, diabetes mellitus, urban living, and sleeping in the same house as an index case. Factors associated with LTBI were increasing age and male gender. Conclusions: Screening for HHC including CXR and TST/IGRA yielded a moderate prevalence of TB disease and infection.

## 1. Introduction

TB in Indonesia is a prominent public health problem and the country has been classified as a high-burden country for tuberculosis [1]. In 2021, Indonesia was the country with the second-highest number of incident cases and accounted for 9.2% of all incident cases globally [1]. The gap between incidence and the detection of TB cases in Indonesia is a particular concern: in 2021, it was estimated that 969,000 (872,000–1,070,000) people in Indonesia fell ill with TB but only 432,577 TB cases were notified, and treatment coverage was an estimated 45% [1]. Moreover, Indonesia’s TB notifications in 2021 fell relative to 2019, likely due to the impact of COVID-19 [1]. The gap between incidence and notified cases is an important issue for Indonesia’s TB response because cases that are not diagnosed and do not commence treatment are likely to be a significant source of TB transmission in the community [2].

A key component of strategies to find missing cases of TB is active case finding (ACF), wherein screening and diagnosis for TB are conducted outside of health facilities to find cases among persons who may not present promptly to a health facility for diagnosis. Contact investigation is considered an effective method for active case finding and involves identifying and screening persons who have had contact with someone known to have TB. Contact investigation has been widely recommended and has two functions, namely, increasing case finding and preventing TB transmission in the community [3]. Therefore, contact investigation has a dual role to diagnose and treat those with active TB disease and to diagnose latent TB infection (LTBI) and provide opportunities for implementing TB preventive treatment. Indonesia’s guidelines recognize the importance of contact investigation and, since 2019, have recommended that contact investigation and TB preventive treatment be performed for all HHCs of bacteriologically confirmed cases [3]. Despite these recommendations, the implementation of household contact investigation in Indonesia in 2022 only achieved a coverage of 13% [4].

Importantly, improving household contact investigation in Indonesia is not solely a question of coverage. The choice of models and algorithms for household contact investigation are also a critical consideration. Indonesia’s current approach to contact investigation is to conduct a home visit, during which HHCs are screened for TB symptoms. Those who have TB symptoms are then referred to a health facility for further investigation for TB and there is variability between health facilities as to the investigations that will be available. The World Health Organization (WHO) recommends using either symptom screen, chest X-ray (CXR), or molecular rapid diagnostic tests alone or in combination with systematic TB screening in HHCs, noting that CXR has high sensitivity for detecting TB disease, especially for sub-clinical TB. However, current WHO recommendations are based on a very low certainty of evidence [5]. A systematic review of the effectiveness of contact tracing in the control of infectious diseases concluded that there is limited evidence of best practices for contact tracing, highlighting the need for further program evaluations [6].

The questions of best practices for contact tracing are applicable for Indonesia, including the potential role and benefit of using CXR. At present, CXR is not recommended for household contact investigation in Indonesia, and there are limited data on the yield and feasibility of incorporating CXR within HHC investigation. However, as Indonesia is expanding TB preventive treatment (TPT) to include HHCs over the age of 5, evidence for optimal HHC screening approaches is needed. This study aimed to describe the diagnostic yield of active TB disease and LTBI among HHCs aged five years and above investigated using CXR-based active case finding and to describe factors associated with active TB and LTBI.

## 2. Materials and Methods

### 2.1. Study Design

This study is part of the Zero TB Initiative Yogyakarta study, an observational prospective cohort study of persons screened using comprehensive mobile TB services integrated in conjunction with contact investigation activities. In June 2020, the Zero TB Yogyakarta project under the Centre of Tropical Medicine, Faculty of Medicine, Public Health and Nursing, Universitas Gadjah Mada, initiated the acceleration of TB elimination in Yogyakarta through a comprehensive approach of “search, treat and prevent”. One of the main community-based ACF strategies used in the Zero TB Project is HHC investigation, in which health workers visit homes of patients with newly identified TB to screen the contacts for TB and refer at-risk individuals to clinics for evaluation and treatment. As distinct from standard HHC investigation in Indonesia, the Zero TB project includes CXR screening and testing for TB infection in its HHC investigation algorithm.

### 2.2. Setting

This study was conducted in two districts, Yogyakarta and Kulon Progo, from 1 June 2020 to 31 December 2022. Yogyakarta city is an urban area with 373,589 inhabitants and a population density of 11,000/km^2^, around fifteen times higher than Kulon Progo (740/km^2^) which is a rural district with a total population of 436,395 [7]. There are 39 primary health centers (Puskesmas) in these districts.

### 2.3. Participants

This study included HHCs of bacteriologically confirmed index cases registered in the Indonesia TB Information system between 1 January 2018 and 31 December 2022. A HHC was defined as an individual who had resided in the same house with an index case for at least one night or for frequent or extended periods during the day with the index case during 3 months prior to the diagnosis of TB [3,8]. HHCs were included in this study if they were aged 5 years or older; if they were domiciled in the Yogyakarta and Kulon Progo districts; and if they were not on TB treatment at the time of contact investigation. Routine contact investigation was conducted among household contacts under the age of 5. However, they were excluded from this study because testing for TB infection was not performed in asymptomatic child contacts. HHCs with a history of TB treatment were included in the study but were excluded from analysis of LTBI diagnosis.

### 2.4. Study Procedures and Data Collection

**Figure 1:**
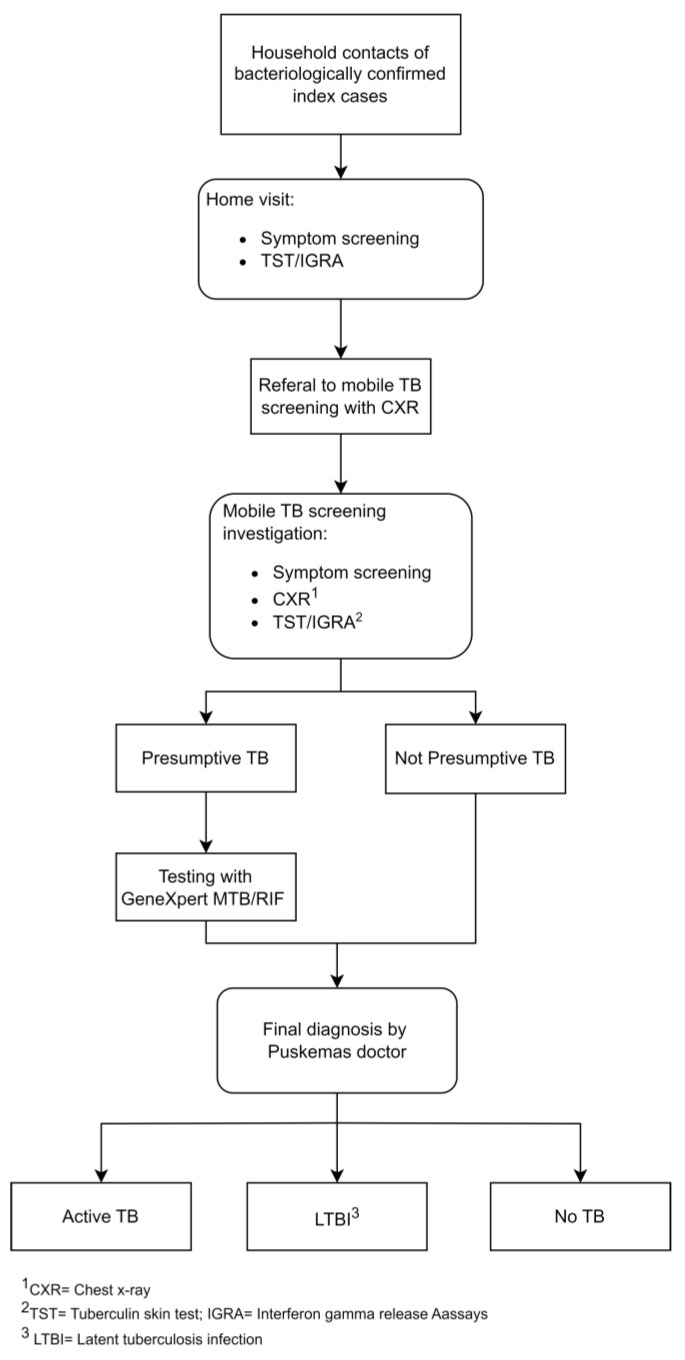
Algorithm of household contact investigation integrated with mobile chest X-ray screening.

The contact investigation algorithm is shown in Figure 1. Details of index cases and their HHCs were obtained by the Zero TB project from each participating Puskesmas. The TB officer/nurse in each Puskesmas then made a schedule for conducting home visits to the households of index cases based on the agreement and availability of the index case. A dedicated HHC investigation nurse from the Zero TB project jointly conducted the home visits with the TB officer/nurse. During the home visits, the Zero TB nurses screened the HHCs for TB symptoms. They also performed a TST or took a venous blood sample for interferon gamma release assay (IGRA; which was available temporarily in the project for a minority of contacts) to all eligible HHCs who provided written consent. For IGRA, the blood was sent to a referral hospital, with a result available within five days. TST was read 48–72 h after administration, either at the Puskesmas or at home by a nurse. HHCs were then given a referral letter to undergo further investigation for TB in a mobile TB service (including CXR for screening) in their area.

At the mobile screening site, HHC’s demographic data (age, gender, education), risk factors (history of TB, diabetes mellitus, HIV, smoking), duration of exposure and closeness to the TB index case, as well as anthropometric details (weight, height, body mass index [BMI]) were collected. All eligible HHCs (not pregnant; able to stand; provided written consent) underwent CXR. Those who were positive on symptom screening or had a positive CXR result were assessed as presumptive for TB. Persons aged 15 years or older were positive on symptom screening if they reported coughing for more than two weeks or hemoptysis. Persons under the age of 15 were positive on symptom screening if they reported coughing for more than 2 weeks, hemoptysis, fever for more than 2 weeks, or unexplained weight loss. A single spot sputum specimen was collected from presumptive cases at the mobile screening site or at home for those who could not produce sputum on site. The sputum was then sent to the nearest Puskesmas or hospital for GeneXpert MTB/RIF testing. TST was performed by a trained nurse at the mobile screening site for those HHCs who had not already had a TST performed during the home visit. Nutritional status for adult participants was assessed based on their BMI: underweight (BMI values < 18.5); normal or desirable weight (BMI values 18.5–22.9); overweight (BMI values ≥ 23, taking into account new consensus on BMI cut points for Southeast Asia, which are similar to BMI cut points in routine use in Indonesia [9]).

All data (symptoms, CXR, TST, and Xpert/MTB RIF) were provided to the doctors in the Puskesmas during clinical review, and the Puskesmas doctor decided the final diagnosis (either bacteriologically confirmed TB, clinical TB, LTBI, or none) and further management, including provision of TB preventive treatment (TPT). The pulmonologist and pediatric TB consultants from Zero TB team provided consultation for the Puskesmas doctors to support diagnosis and management for cases where there were management questions or doubt. Clinical TB was established based on assessment of the combined clinical picture of history, examination, and radiological and microbiological findings. Latent TB infection was defined as those who had no TB disease and the result of TST (transversal induration of ≥10 mm) or if IGRA was positive. HHCs were followed up for 1 year to check for possible progression to active TB.

### 2.5. Variables

All data were collected using REDCap electronic data capture tools hosted by the Universitas Gadjah Mada [10]. Data were then imported into STATA17 (StataCorp, College Station, TX, USA) for data cleaning and analysis. Data were checked for data sanity. Potential errors were identified and cleaned according to pre-specified rules in the data cleaning and analysis plan. Categorical variables including outcomes for the diagnostic cascades were presented as frequency and proportions, whereas continuous variables were presented as mean and standard deviations. Associations between person characteristics and active TB and LTBI were analyzed using univariate and multivariate logistic regression.

## 3. Results

### 3.1. Active TB Cases Cascade among Household Contacts

From 1196 index cases with bacteriologically confirmed TB, 2857 HHCs were included in this study. The mean age of the HHCs was 44 years (±20.5), with 55.9% (1598/2857) female. A total of 15.9% (455/2857) HHCs screened positive for presumptive TB. The yield of active TB among the HHCs was 2.4% (68/2857) for TB and 0.5% (13/2857) for bacteriologically confirmed TB. If CXR had not been performed, then symptom screening would have identified 7.6% (216/2857) of contacts as having presumptive TB contacts and would have given a yield of 0.2% (5/2857) for bacteriologically confirmed TB (Table 1).

### 3.2. Latent TB Infection Cascade among Household Contact

There were 2621 eligible HHCs screened for LTBI. Of these, 236 HHCs were not tested for LTBI due to active TB (n = 68) or a past history of TB treatment (n = 168). The percentage of HHCs positive for LTBI was 45.7% (1083/2371) (Table 2).

### 3.3. Factors Associated with Active TB Disease among Household Contacts

There were 68 cases of all forms of TB, with a mean age of 44.1 years (±20.5) and 48.5% (33/68) were female. In multivariate logistic regression, factors associated with increased odds of active TB disease were increasing age (aOR 1.02, 95%CI 1.01–1.03), being underweight (aOR 2.52, 95%CI 1.42–4.47), diabetes mellitus (aOR 2.60, 95%CI 1.08–6.24), residing in an urban district (aOR 1.86, 95%CI 1.07–3.22), and sleeping in the same house as the TB index case (aOR 5.29, 95%CI 1.28–21.89); while being overweight was associated with lower odds of being diagnosed with active TB (aOR 0.37, 95%CI 0.18–0.73) (Table 3).

### 3.4. Factors Associated with Latent TB Infection among Household Contacts

From the 1083 HHCs diagnosed with LTBI, the mean age was 40.62 (±19.42) and 54.1% (586/1083) were female. In a multivariate analysis, increasing age was associated with increased odds of LTBI (aOR 1.01, 95%CI 1.01–1.02), while being female (aOR 0.77, 95%CI 0.61–0.97) was associated with lower odds of LTBI (Table 4).

## 4. Discussion

Our study identified a yield of active TB disease of 2.4% amongst HHCs of bacteriologically confirmed TB index cases who were screened using chest X-ray. The yields from HHC investigations reported in other studies are varied. Our result is similar to that documented in a systematic review of contact investigation amongst contacts of smear-positive index patients in low/middle-income countries, which was 3.1% (95% CI 2.1–4.5) [11]. Our yield was higher than that reported from Nepal (1.6%) [12], Vietnam (1.0%) [13], India (1.15%) [14], and Iran (1.1%) [15]. Much higher yields have been reported from Pakistan (15.9%) [16], South India (5.3%) [17], and Tanzania (6.4%) [18]. The variation in yield might be related to differences in the study populations, study settings including TB rates, household ventilation and sleeping arrangements, and study methodology, with differences in sample size, method of TB screening, and diagnostic criteria.

The yield in our study may have been influenced by the inclusion of HHC investigations for index cases from a preceding 3-year period. Incidence of new cases amongst HHCs is highest in the first year after contact with an index case and remains above the background incidence for at least 5 years after exposure to a patient with TB [11]. Because some of our contact investigations took place more than one year after HHCs had contact with the index case, there may have been TB cases diagnosed among HHCs after contact but before our HHC investigation. This would have lowered the yield of our HHC investigation relative to an approach where HHC investigation is always undertaken more proximally to the time the index case is notified.

In our study, we found a high uptake for screening with CXR (82.9%) and testing for TB infection (90%) among HHCs. Although considerable guidance for contact investigation is provided by national policies, implementation takes place locally [19]. Within a country there are considerable differences and a variety of circumstances that could influence the way contact investigation is most effectively conducted. Engaging full-time, salaried community health workers in TB active-case finding schemes can lead to greater impact for both active TB and LTBI [20,21], and this human resource model should be prioritized for scale up where resources permit [22]. In this study, we used a dedicated nurse from the community to conduct the TB screening and LTBI testing during home visits. Inclusion of dedicated nurses with competence in community engagement can help to overcome problems regarding the acceptance of index cases and contacts to be screened during home visits and to mobilize contacts for CXR screening. In addition, they can carry out TST examinations during home visits, with positive results potentially increasing the willingness of contacts to undergo CXR screening. This study contributes to the growing literature documenting the impact of community-based ACF on increasing TB case notifications. Similarly, a study in Vietnam conducted mobile ACF closer to the sub-district location, increasing CXR accessibility and service uptake through collaborating with subdistrict health facilities to mobilize contacts to attend screening [13].

The yield of LTBI amongst HHCs in our study was 45.7%. This is similar to systematic review findings by Velleca et al., who reported a 45% (95% CI 35–55) yield amongst contacts of TB index cases [23], and comparable with studies from South Asia [24,25]. However, a study in Semarang, Indonesia, found a much higher proportion of 63.8% amongst HHCs. Differences in LTBI yield might be due to the study inclusion criteria, with our study including children and adolescents aged between 5 and 15 who may have a lower baseline rate of LTBI prior to household exposure compared with older adults [26].

The inclusion of CXR within the TB screening algorithm likely increased the yield of active TB detection among HHCs. In this study, the proportion of presumptive TB among HHCs was 15.9%, compared to 7.6% if symptom screening alone had been used. Adding CXR to TB screening resulted in the detection of 0.5% bacteriologically confirmed TB versus 0.2% if only symptom screening had been done. Arguably, the bigger difference was the identification of sub-clinical TB. Without CXR for the screening of presumptive TB, 9 of the 13 bacteriologically confirmed cases and, potentially, 55 clinically diagnosed cases would have been missed, which included both symptomatic and asymptomatic cases. It is still possible that the inclusion of CXR increases the risk of overdiagnosis of TB. However, a recent review reported that between 36 and 80% of prevalent bacteriologically confirmed TB is subclinical, which is consistent with our results [27]. The inclusion of children aged 5–10, who may be more likely to be bacteriologically negative on sputum testing, may also have affected the proportion of bacteriologically confirmed cases in this study.

Indonesia has recently revised guidelines to include all HHCs regardless of age to be included for systematic screening and to be eligible for TB preventive treatment. However, currently the program does not include offering CXR screening for all HHCs. Our study suggests that screening HHCs without CXR will miss significant numbers of active TB cases. In addition, it is likely that TB preventive treatment will be offered to some of these missed cases with the potential for undertreatment and amplification of resistance. This study has shown that inclusion of CXR within HHC systematic screening is feasible, with high uptake of the offered services. The scale up of TB active case finding with increased availability of mobile CXR in Indonesia provides an opportunity to strengthen the HHC training system within Indonesia. Our findings support the recommendation of the use of CXR in combination with symptoms for HHC screening for better case finding.

Our study an identified increasing age of HHCs as being associated with LTBI when adjusting for other factors. This might be due to the fact that elderly caregivers are more likely to spend a longer duration providing TB care to index patients than their younger counterparts, who usually go to work [28]. It may also reflect historically higher rates of TB in the past or simply that with increasing age there is greater time to be exposed and infected with TB in an endemic setting. This finding is consistent with the literature [24,29]. This study also identified male sex as being associated with LTBI. This is in line with previous studies conducted in South Korea and the United Kingdom [30,31]. Factors that contribute to this difference have been suggested to be the risk-taking behaviors of males, sociocultural factors, genetic predisposition, and immunological attributes [32,33].

This study has a number of limitations. We retrospectively estimated TB diagnosis that likely would have been missed if CXR had not been used, which may have over- or under-estimated how a symptom screening and geneXpert molecular diagnosis algorithm would have performed. Clinical diagnosis was based on CXR abnormality, which involved CXR reading by multiple different readers, which could lead to overdiagnosis. However, the support provided by a pulmonologist and a pediatrician who were available to discuss cases likely reduced this risk. The diagnosis of LTBI was based on the TST cut off of 10 mm, which has been commonly used in practice in Indonesia for HHCs. Recently, Indonesia has changed the guidelines to be in line with the WHO guidelines, to a cut off of ≥5 mm for HHCs [34]. This would have resulted in an extra 197 HHCs diagnosed with LTBI and could potentially have changed associations. The strengths of this study included the prospective data collection and the large numbers of HHCs screened.

## 5. Conclusions

The yield of TB in HHCs of bacteriologically confirmed TB cases, who were screened using symptoms and chest X-ray, in two districts of the Yogyakarta province was 2.4% for active TB and 45.7% for LTBI. The inclusion of CXR within the screening algorithm likely greatly increased the yield of bacteriologically confirmed TB cases, as well as clinically diagnosed cases. This study suggests that the current approach to household contact screening in Indonesia based on symptom screening alone will miss a large proportion of active TB disease and argues for the inclusion of CXR within Indonesia’s TB household contact investigation program.

## Figures and Tables

**Table 1:** Active TB case cascade among household contacts.

Variable	Symptom and CXR Screening + Bacteriological Testing N (%)	Symptom Screening Only + Bacteriological Testing N (%)
HHC symptoms screened	2857 (100%)	2857 (100%)
HHC CXR screened	2368 (82.9%)	-
HHC presumptive TB	455 (15.9%)	216 (7.6%)
HHC diagnosed TB	68 (2.4%)	5 (0.2%)
HHC bacteriologically confirmed	13 (0.5%)	5 (0.2%)

Note: HHC = household contact, CXR = Chest X-ray, TB = tuberculosis.

**Table 2:** Latent TB infection cascade among household contacts.

Variable	Total N = 2857 (%)
Household contacts eligible for LTBI screening	2621 (92.0%)
Household contacts tested for LTBI	2371 (90.5%)
Tested with IGRA	359 (15.1%)
Tested with TST	2012 (84.9%)
Household contacts positive for LTBI	1083 (45.7%)
Positive IGRA result	124 (5.2%)
Positive TST result	959 (40.4%)

Note: LTBI = Latent tuberculosis infection.

**Table 3:** Factors associated with active TB disease among household contacts.

Summary Statistics					Univariate			Multivariate		
Characteristic	No TB Disease N = 2789	Active TB Disease N = 68 ^1^	Total N = 2857	*p*-Value	OR ^2^	95% CI ^2^	*p*-Value	aOR ^2^	95% CI ^2^	*p*-Value
**Age (years)**	37.6 (20.4)	44.1 (20.5)	37.7 (20.4)		1.02	(1.00, 1.03)	0.0093	1.02	[1.00, 1.03]	0.0015
**Sex**										
Male	1224 (43.9%)	35 (51.5%)	1259 (44.1%)	0.21	1.00			1.00		
Female	1565 (56.1%)	33 (48.5%)	1598 (55.9%)		0.74	(0.46, 1.19)	0.2149	1.07	[0.53, 2.12]	0.8572
**Nutritional status**										
Normal weight	856 (35.8%)	25 (37.9%)	881 (35.8%)	<0.001	1.00			1.00		
Underweight	526 (22.0%)	28 (42.4%)	554 (22.5%)		1.82	(1.05, 3.16)	0.0325	2.52	[1.42, 4.47]	0.0016
Overweight	1012 (42.3%)	13 (19.7%)	1025 (41.7%)		0.44	(0.22, 0.87)	0.0173	0.37	[0.18, 0.73]	0.0046
**Smoker**										
No	2140 (76.7%)	43 (63.2%)	2183 (76.4%)	0.010	1.00			1.00		
Yes	649 (23.3%)	25 (36.8%)	674 (23.6%)		1.92	(1.16, 3.16)	0.0108	1.81	[0.88, 3.74]	0.1076
**Diabetes mellitus status**										
No or Unknown	2674 (95.9%)	61 (89.7%)	2735 (95.7%)	0.013	1.00			1.00		
Yes	115 (4.1%)	7 (10.3%)	122 (4.3%)		2.67	(1.19, 5.96)	0.0167	2.60	[1.08, 6.24]	0.0324
**District type**										
Rural district	1011 (36.2%)	20 (29.4%)	1031 (36.1%)	0.25	1.00			1.00		
Urban district	1778 (63.8%)	48 (70.6%)	1826 (63.9%)		1.36	(0.81, 2.31)	0.2478	1.86	[1.07, 3.22]	0.0274
**Type of contact**										
Did not sleep in the same house	384 (13.8%)	2 (2.9%)	386 (13.5%)	0.010	1.00			1.00		
Slept in same house	2405 (86.2%)	66 (97.1%)	2471(86.5%)		5.27	(1.29, 21.60)	0.0210	5.29	[1.28, 21.89]	0.0217
**Time since last TB contact**										
<1year	2385 (85.5%)	58 (85.3%)	2443 (85.5%)	0.21	1.00			1.00		
≥1 year	404 (14.5%)	10 (14.7%)	414 (14.5%)		0.87	(0.52, 2.01)	0.9593	0.87	[0.43, 1.75]	0.6978

^1^ N (%); ^2^ OR = Odds Ratio, CI= Confidence Interval, aOR = adjusted OR.

**Table 4:** Factors associated with latent TB infection among household contacts.

Summary Statistics					Univariate			Multivariate		
Characteristic	No TB Infection N = 1288 ^1^	TB Infection N = 1083 ^1^	Total N = 2371	*p*-Value	OR ^2^	95% CI ^2^	*p*-Value	aOR ^2^	95% CI ^2^	*p*-Value
**Age (years)**	36.20 (20.86)	40.62 (19.42)	38.22 (20.33)	<0.001	1.01	(1.00, 1.03)	0.0000	1.01	[1.01, 1.02]	0.0000
**Sex**										
Male	517 (40.1%)	497 (45.9%)	1014 (42.8%)	0.005	1.00			1.00		
Female	771 (59.9%)	586 (54.1%)	1357 (57.2%)		0.79	(0.67, 0.93)	0.0048	0.07	[0.61, 0.97]	0.0248
**Nutritional status**										
Normal weight	405 (35.4%)	337 (36.1%)	742 (35.7%)	0.012	1.00			1.00		
Underweight	266 (23.2%)	169 (18.1%)	435 (20.9%)		0.76	(0.60, 0.97)	0.0282	0.89	[0.69, 1.15]	0.3729
Overweight	474 (41.4%)	428 (45.8%)	902 (43.4%)		1.09	(0.89, 1.32)	0.4111	1.06	[0.87, 1.29]	0.5679
**Smoker**										
No	1018 (79.0%)	801 (74.2%)	1819 (76.7%)	0.004	1.00			1.00		
Yes	270 (21.0%)	282 (26.0%)	552 (23.3%)		1.33	(1.10, 1.61)	0.036	1.06	[0.81, 1.37]	0.6887
**Diabetic status**										
No or Unknown	1239(96.2%)	1038 (95.8%)	2277 (96.0%)	0.66	1.00			1.00		
Yes	49 (3.8%)	45 (4.2%)	94 (4.0%)		1.10	(0.73, 1.66)	0.6629	0.80	[0.51, 1.24]	0.3043
**District type**										
Rural district	501 (38.9%)	355 (32.8%)	856 (36.1%)	0.002	1.00			1.00		
Urban district	787 (61.1%)	728 (67.2%)	1515 (63.9%)		1.31	(1.10, 1.55)	0.0020	1.18	[0.99, 1.42]	0.0318
**Type of contact**										
Did not sleep in the same house	178 (13.8%)	140 (12.9%)	318 (13.4%)	0.53	1.00			1.00		
Slept in same house	1110(86.2%)	943 (87.1%)	2053 (86.4%)		1.08	(0.85, 1.37)	0.5252	1.08	[0.84, 1.40]	0.5421
**Time since last TB contact**										
<1 year	1102 (85.6%)	946 (87.3%)	2048 (86.4%)	0.21	1.00			1.00		
≥1 year	186 (14.4%)	137 (12.7%)	323 (13.6%)		0.86	(0.68, 1.09)	0.2057	0.84	[0.66, 1.08]	0.1817

^1^ N (%); ^2^ OR = Odds Ratio, CI = Confidence Interval, aOR = adjusted OR.

## Data Availability

Due to data privacy concerns, the data are not publicly available. However, reasonable data requests may be granted through contacting the corresponding author.
